# Left bundle branch area pacing for the treatment of painful left bundle branch block syndrome

**DOI:** 10.1016/j.hrcr.2022.12.003

**Published:** 2022-12-09

**Authors:** Maxime Cerantola, David S. Frankel, David J. Callans, Pasquale Santangeli, Robert D. Schaller

**Affiliations:** Electrophysiology Section, Cardiovascular Division, Perelman School of Medicine at the University of Pennsylvania, Philadelphia, Pennsylvania

**Keywords:** Painful left bundle branch block, Deep septal pacing, Dyssynchrony, Chest pain, Cardiac resynchronization

## Introduction

Painful left bundle branch block (LBBB) syndrome causes intermittent or chronic chest pain and/or shortness of breath in the absence of myocardial ischemia.^1^ Given its low prevalence and association with coronary artery disease, it is frequently not recognized, making its true prevalence unknown. The mechanistic nature of the associated chest pain has not been completely elucidated but is thought to be related to ventricular dyssynchrony^2^ and interoceptive hypersensitivity.^3^ Treatment options include the use of beta-blockers to reduce heart rate and cardiac pacing therapy aimed at reestablishing normal ventricular activation, through biventricular or His bundle pacing (HBP).^1^^,^^4^, ^5^, ^6^, ^7^ HBP can be challenging and is associated with high pacing thresholds, low sensing, and the need for late lead revisions.^8^ Left bundle branch area pacing (LBBAP) is an emerging technique with similar benefits to HBP and has been described in the setting of painful LBBB syndrome.^9^ Herein, we describe a series of patients with painful LBBB syndrome treated with LBBAP.

## Case report

### Case 1

**Figure 1:**
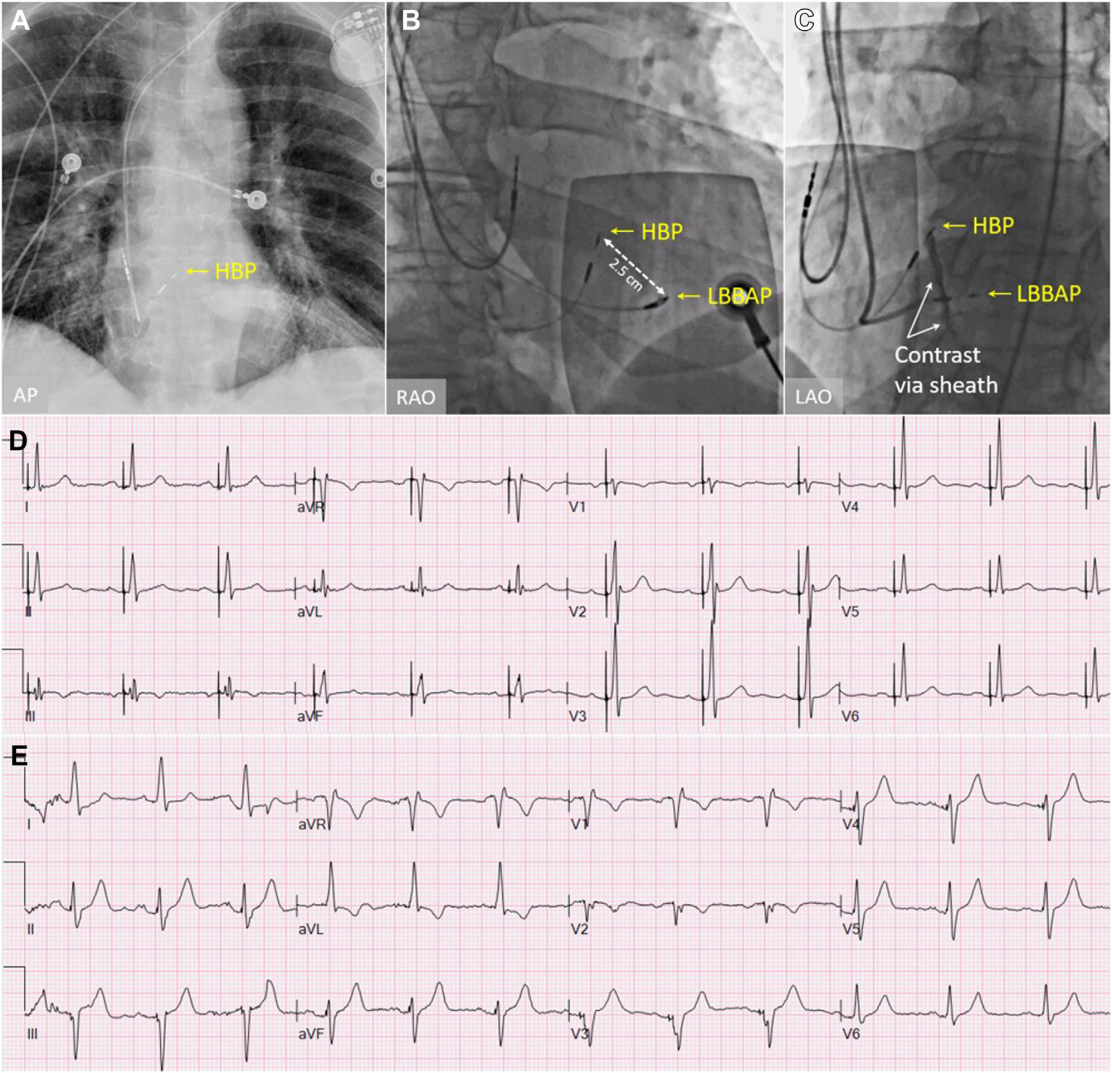
**A:** Chest radiograph showing a dual-chamber pacemaker with a His bundle lead. **B:** Fluoroscopic image showing the location of the left bundle branch area pacing (LBBAP) lead in relation to the His bundle pacing (HBP) lead prior to placement. **C:** Contrast injection through the implantation sheath confirms the tip of the LBBAP lead to be in the deep septum. **D:** A 12-lead electrocardiogram (ECG) showing selective His bundle capture. **E:** A 12-lead ECG of the same patient with LBBAP and a slightly shorter stimulus-to-peak left ventricular activation time (LVAT) in lead V_6_. Note the His bundle lead is pacing 40 ms after the LBBAP lead. AP = anterior-posterior; LAO = left anterior oblique; RAO = right anterior oblique.

A 65-year-old man with intermittent exertional chest pain and heaviness was diagnosed with painful LBBB syndrome and underwent therapeutic HBP (Figure 1A and 1D).^4^ His capture threshold with LBBB correction was 2.5 V @ 1 ms with R-wave sensing of 2.1 mV and bipolar impedance of 777 ohms. Rapid battery depletion ensued as the patient progressed to complete heart block, reaching elective replacement interval at 4 years. Upgrade to a biventricular pacemaker with the addition of an LBBAP lead was recommended to increase battery longevity.

The patient presented to the electrophysiology laboratory in a fasting state and conscious sedation was used to maintain verbal communication during the procedure. A 7F hemostatic peel-away sheath (SafeSheath; Pressure Products, San Pedro, CA) was placed in the left axillary vein, through which a nondeflectable 7F sheath (C315; Medtronic, Minneapolis, MN) was guided to the right ventricular (RV) septum. A 4.1F bipolar, steroid-coated lead (Select Secure 3830; Medtronic) was placed in the delivery sheath with the tip 2–3 cm inferoapical to the pre-existing HBP lead tip, using only an anatomical approach (Figure 1C). Pacing of the RV septum as well as sheath-induced premature ventricular complexes elicited chest discomfort, confirming again painful LBBB syndrome. Maintaining the sheath on the septum and facing toward 2 o’clock in a right anterior oblique fluoroscopic angle of 20 degrees, the lead was screwed into the myocardium until the ring exited the sheath and fixation beats^10^ were appreciated.

Contrast injection through the sheath confirmed a deep septal position (Figure 1C). Unipolar pacing showed a QRS complex of 140 ms with a terminal r wave in lead V_1_, R waves of 11 mV, and a stimulus-to-peak left ventricular activation time in V_6_ (LVAT) of 71 ms (Figure 1E), slightly shorter than HBP (Figure 1D). The unipolar pacing threshold was 0.4 V @ 0.4 ms with an impedance of 665 ohms. Bipolar pacing similarly showed a threshold of 0.4 V @ 0.4 ms with an anodal stimulation threshold of 4V @ 0.4 ms. No LBB potential was seen when pacing from the pre-existing HBP lead.

LBBAP resulted in no chest pain in any configuration, including during anodal capture. The lead was secured and plugged into the right ventricular port and the chronic HBP lead was left in place and plugged into the left ventricular port of a biventricular pacemaker (LBBAP 40 ms early). At device follow-up, the LBBAP lead showed stable parameters, at which point the HBP lead was turned off. The patient remains asymptomatic at 12 months.

### Case 2

**Figure 2:**
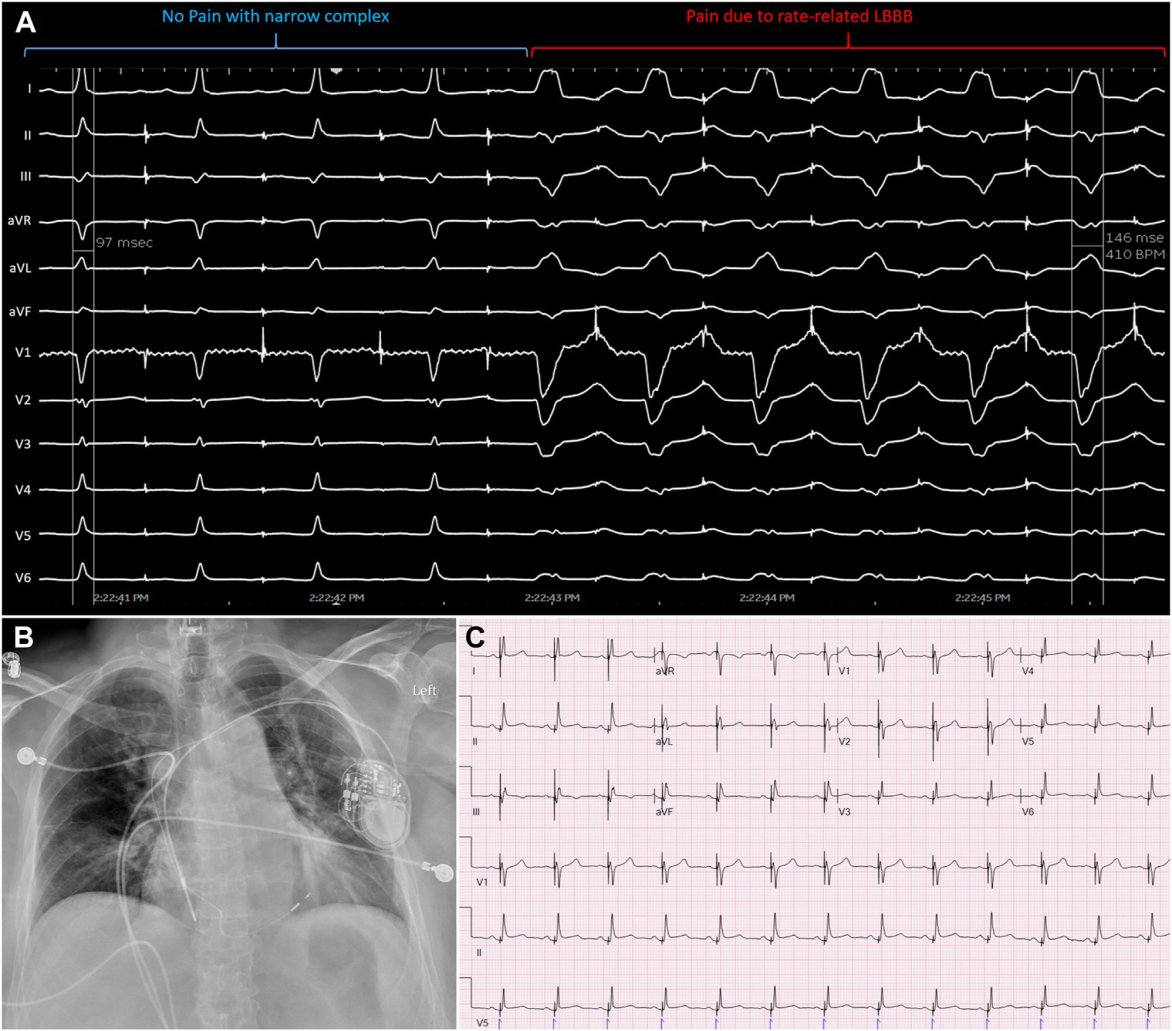
**A:** Atrial pacing shows development of a left bundle branch block (LBBB) at slightly faster rates than sinus, associated with chest pain. **B:** Chest radiograph of a dual-chamber pacemaker with a left bundle branch area pacing (LBBAP) lead. **C:** LBBAP 12-lead electrocardiogram morphology. Note the lack of a terminal R wave in lead V_1_ owing to fusion with native conduction.

A 58-year-old woman with a history of hypertension and symptomatic paroxysmal supraventricular tachycardia was found to have intermittent LBBB above heart rates of 100 beats/min after slow pathway modification. Aberrancy was associated with chest pain, which immediately resolved upon administration of intravenous metoprolol, resulting in heart rate slowing and loss of aberrancy. A diagnosis of painful LBBB syndrome was made and a dual-chamber pacemaker with an LBBAP lead was recommended. The patient was prepped in a similar fashion and conscious sedation was used to maintain verbal communication during the procedure. A 7F hemostatic peel-away sheath (SafeSheath) was placed in the left axillary vein, through which an atrial pacing lead was placed. Atrial pacing at a rate of 100 beats/min resulted in rate-related LBBB and pain (Figure 2A). LBBAP in a bipolar configuration showed a QRS complex of 132 ms with a small terminal R wave in V_1_ with an anodal threshold of 2.4 V @ 0.5 ms and a cathodal threshold of 0.75 V @ 0.4 ms, R waves of 20 mV, impedance of 475 ohms, and an LVAT of 74 ms (Figure 2B and 2C). Lead parameters were stable upon follow-up and the patient remains symptom free at 8 months.

### Case 3

**Figure 3:**
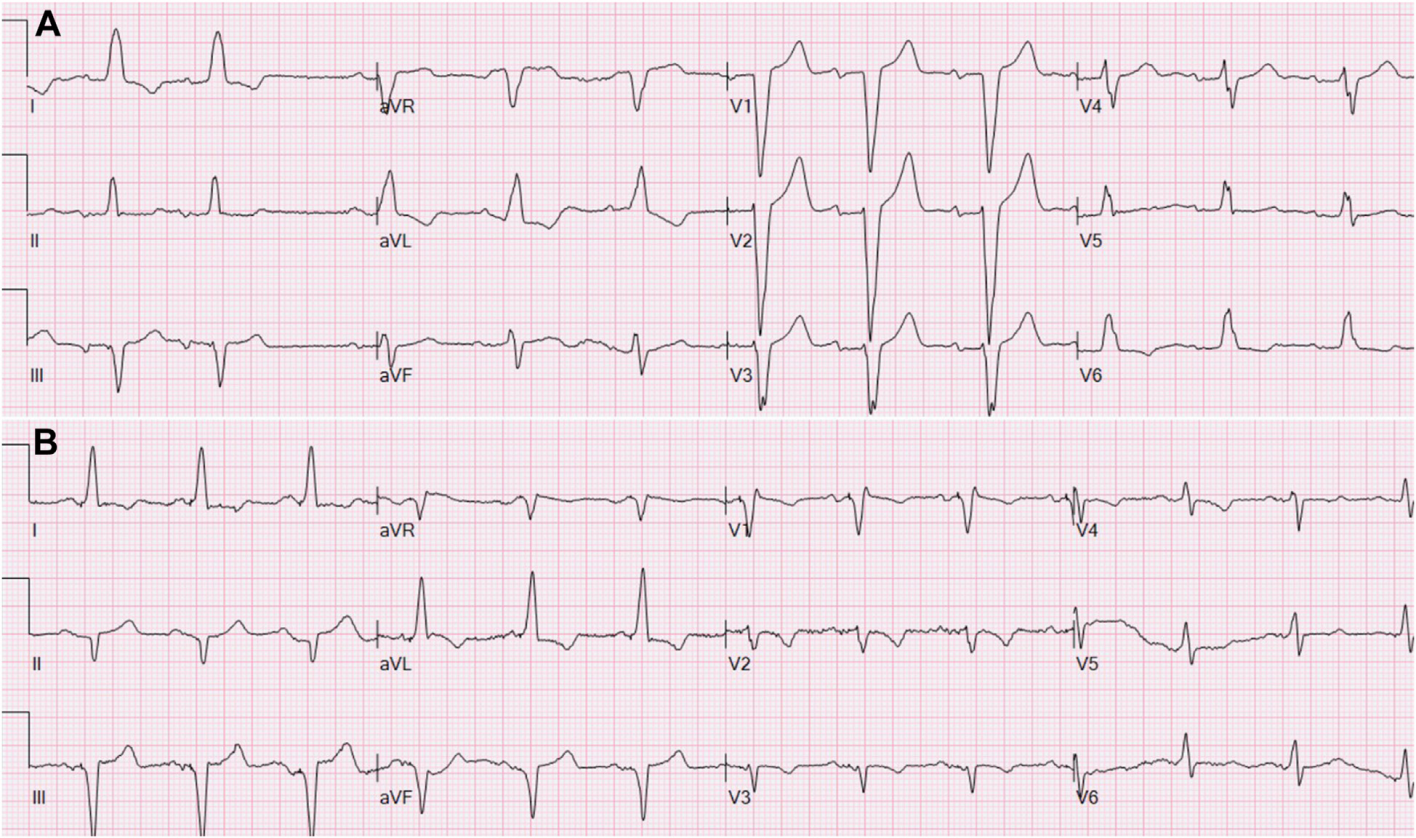
**A:** A 12-lead electrocardiogram (ECG) of baseline left bundle branch block associated with chronic pain. **B:** A 12-lead ECG of left bundle branch area pacing without pain.

An 82-year-old man with a history of coronary artery disease and percutaneous coronary intervention 2 decades prior was noted to have intermittent LBBB associated with exercise intolerance upon outpatient monitoring. An exercise stress test confirmed LBBB 3 minutes into exertion with associated shortness of breath. Coronary angiography did not demonstrate any new obstructive coronary lesions. Over the subsequent 4 months, his shortness of breath became constant, with limitations of daily activities, and LBBB was found to be present at resting heart rates (Figure 3A). Given the association of LBBB with his symptoms, LBBAP was recommended and placed as previously described. The bipolar pacing threshold was 0.4 V @ 0.4 ms, with R waves of 7 mV, impedance of 665 ohms, and an LVAT of 74 ms (Figure 2B). Lead parameters were stable upon follow-up and the patient remains symptom free at 6 months.

## Discussion

Painful LBBB syndrome is a rare entity that is likely under-reported,^1^ and HBP has been shown to be an effective long-term solution for this challenging disease.^4^, ^5^, ^6^ Similarly, HBP has also been shown to provide effective relief with painful RV pacing.^11^ However, acute procedural challenges and long-term suboptimal parameters have limited its appeal and made way for the emergence of LBBAP, a more appealing conduction system pacing technique owing to its high procedural success, low and stable capture thresholds, and excellent sensing.^12^ In a previous report by Garg and colleagues,^9^ 2 patients underwent successful LBBAP for painful LBBB syndrome with clear retrograde LBB potentials seen during RV pacing.^9^ Although the terminal R wave in lead V_1_ as well as the rapid LVAT suggest that the left-sided conduction system was being recruited in our cohort, no LBB potentials or output-dependent changes were seen. Although LBBAP criteria are still evolving,^13^ it is possible that the left septum was being paced without recruitment of the LBB in some or all of these patients. While stricter criteria will need to be developed to appropriately judge this strategy, it is encouraging that LBBAP does seem to be forgiving in the context of the painful LBBB syndrome, as all leads were placed using an anatomical approach targeting the region inferior and apical to the His bundle. This is consistent with prior studies that have shown that pacing of the left side of the ventricular septum results in a similar activation pattern as seen in normal sinus rhythm and can increase cardiac function.^14^^,^^15^ Maintenance of direct verbal communication with the patient during the procedure is critical to ensure that the pacing location is therapeutic. Although all patients in this series were programmed to fixed AV delays, dynamic delays can be considered in patients with intermittent LBBB, which requires further study.

## Conclusion

Painful LBBB syndrome should be considered in patients with chest pain, shortness of breath, and intermittent or chronic LBBB, without other identified causes. LBBAP shows excellent pacing parameters, including a low capture threshold, and effectively treated all 3 of our patients despite lack of definitive evidence of LBB capture. LBBAP appears to be as effective as HBP without the limitations of high pacing threshold and frequent need for late lead revision. More data are needed to refine LBB capture criteria and assess the long-term efficacy of this approach.

**Key Teaching Points**

• Painful left bundle branch block syndrome is likely an under-recognized disease that can lead to diagnostic delay and significant morbidity.
• His bundle pacing and left bundle branch area pacing have been shown to be effective therapies for this syndrome by reestablishing normal physiologic activation.
• Left septal pacing appears to provide sufficient resynchronization for resolution of symptoms despite lack of engagement of the conduction system.
